# Copper(II) 2,2-Bis(Hydroxymethyl)Propionate Coordination Compounds with Hexamethylenetetramine: From Mononuclear Complex to One-Dimensional Coordination Polymer

**DOI:** 10.3390/molecules26113358

**Published:** 2021-06-02

**Authors:** Sadaf Rauf, Agata Trzesowska-Kruszynska, Tomasz Sierański, Marcin Świątkowski

**Affiliations:** Institute of General and Ecological Chemistry, Lodz University of Technology, Zeromskiego 116, 90-924 Lodz, Poland; sadaf.rauf@dokt.p.lodz.pl (S.R.); agata.trzesowska@p.lodz.pl (A.T.-K.); tomasz.sieranski@p.lodz.pl (T.S.)

**Keywords:** copper, hexamethylenetetramine, 2,2-bis(hydroxymethyl)propionate, crystal structure, coordination polymer, metal-ligand stoichiometry, UV-Vis, IR, thermal analysis, TD-DFT calculations

## Abstract

Three new copper coordination compounds derived from 2,2-bis(hydroxymethyl)propionic acid (dmpa) and hexamethylenetetramine (hmta) were obtained and their crystal structures were determined. The stoichiometry of the reagents applied in the syntheses reflects the metal to ligand molar ratio in the formed solid products. Due to the multiple coordination modes of the used ligands, wide structural diversity was achieved among synthesized compounds, i.e., mononuclear [Cu(dmp)_2_(hmta)_2_(H_2_O)] (1), dinuclear [Cu_2_(dmp)_4_(hmta)_2_] (2), and 1D coordination polymer [Cu_2_(dmp)_4_(hmta)]_n_ (3). Their supramolecular structures are governed by O—H•••O and O—H•••N hydrogen bonds. The compounds were characterized in terms of absorption (UV-Vis and IR) and thermal properties. The relationships between structural features and properties were discussed in detail. Owing to discrepancies in the coordination mode of a dmp ligand, bidentate chelating in 1, and bidentate bridging in 2 and 3, there is a noticeable change in the position of the bands corresponding to the stretching vibrations of the carboxylate group in the IR spectra. The differences in the structures of the compounds are also reflected in the nature and position of the UV-Vis absorption maxima, which are located at lower wavelengths for 1.

## 1. Introduction

Coordination compounds have attracted interest, not only because of their different topologies and properties but also due to their varied applications. Since the structure has a large impact on the exhibited properties, coordination compounds, especially coordination polymers and metal–organic frameworks, have become a focus of research in the field of crystal engineering [1]. Along with hydrogen bonds, coordination bonds have become interactions that are considered in crystal design [2,3]. Designing a coordination compound with the desired molecular and crystalline properties requires an understanding of the influence of the metal-ligand ratio, ligand functionality, and metal coordination properties on crystal packing. The ability to control the way the molecules are ordered in the solid state could control exhibited properties. In this field, much has been achieved regarding the usage of organic linker ligands to design compounds capable of the absorbtion of gases [4,5], separation of compounds [6,7], or acting as effective catalyst [8,9,10]. The tuneable position of the coordinating groups in these organic spacers enables adequate control over the topology and dimensionality of the resulting networks.

Hexamethylenetetramine (hmta), as a compound with a cage-like structure that adopts different coordination modes starting from terminal monodentate up to tetradentate modes, is considered a simple heterocyclic linker. Although many coordination compounds containing hmta were obtained, problems with controlling both the coordination mode of hmta and the molecular self-assembly remained unsolved. The coordination mode adopted by hmta may depend on the metal ion and co-ligands used to synthesize the coordination compound. The choice of the auxiliary ligand is wide-ranging from simple and small inorganic/organic ions to bulk ions but the prediction of the molecular and crystal structure is difficult because hmta and the co-ligand interplay with each other and they both influence network formation. For example, single inorganic ions like halides, nitrate, or thiocyanate ions, form together with hmta diverse coordination networks, from 1D chains to 3D frameworks [11,12]. The carboxylate ligands usually form coordination compounds with repeating coordination entities extending in three dimensions [11]. There is also a lot of discrete hmta coordination compounds [13].

The main goal of the presented research was to determine the influence of the metal–ligand ratio on the self-assembly of building blocks, including the potentially tetradentate hmta ligand, 2,2-bis(hydroxymethyl)propionic acid (other names: 3-hydroxy-2-(hydroxymethyl)-2-methylpropanoic acid or dimethylol propionic acid; abbreviation: dmpa). Such a combination of ligands should lead to a diversity of obtained structures since the hydroxycarboxylate ions can use the carboxylate group and the hydroxyl group to form coordination bonds. Usually, the *α*-hydroxycarboxylate ligands are used as metal-binding units in coordination networks [14,15,16]. The β-hydroxycarboxylate ligands, such as 2,2-bis(hydroxymethyl)propionate (dmp), have been less investigated although they can also display versatile coordination modes. So far, the structures of only eight coordination compounds containing dmp were reported in The Cambridge Structural Database [17].

## 2. Results and Discussion

### 2.1. Synthesis and Structural Analysis of Copper Coordination Compounds

The reactions between copper(II) 2,2-bis(hydroxymethyl)propionic acid [Cu(dmp)_2_] and hmta carried out in various substrates ratios, led to three different coordination compounds. The copper:hmta ratios in synthesized compounds directly reflect the reaction stoichiometries. While controlling the metal: ligand ratio in the solid state using the reaction stoichiometry of the substrates is generally known, the direct translation of a synthesis ratio into the solid product is not common in the case of hmta coordination compounds [18,19,20].

**Figure 1:**
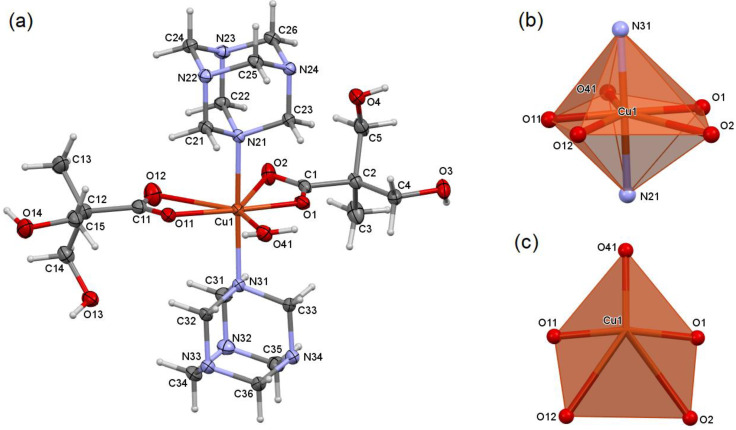
Molecular structure of 1, plotted with a 50% probability of displacement ellipsoids of nonhydrogen atoms and as spheres of arbitrary radii for hydrogen atoms (**a**). Coordination polyhedron of 1, general view (**b**), pentagonal base (**c**).

The mononuclear compound [Cu(dmp)_2_(hmta)_2_(H_2_O)] (1) was formed in the reaction, in which the Cu(dmp)_2_: hmta molar ratio was 1:2. The copper cation is seven-coordinated by two bidentate chelating dmp anions, two monodentate hmta molecules, and one monodentate water (Figure 1a). The coordination polyhedron is a distorted pentagonal bipyramid [21], in which both apexes are occupied by nitrogen atoms of hmta (Figure 1b,c). A strong asymmetricity of coordination bonds between copper ion and chelating dmp anions is observed (Table 1). The steric hindrance of branched dmp anions precludes them from forming two coordination bonds of similar length. The total valence of copper cation (1.823 v.u. [22,23,24,25], Table 1) is significantly below the expected value 2+ (formal oxidative state), which confirms strong strains in the inner coordination sphere. The weaker coordination bonds of chelating dmp anions are ca. 1 Å longer than the stronger bonds. Despite their relatively greater length, they are binding in character, which was confirmed by natural bond orbital (NBO) analysis and an electron density map (Figure S1). Moreover, in similar seven-coordinated copper coordination compounds, comparable or even longer Cu-O coordination bonds of chelating carboxylates were found, e.g., butenedicarboxylate: 2.908 Å in LULSUG (CSD Refcode) [26]; benzenetricarboxylate: 2.979 Å in DUYCOQ [27]; acetate: 3.000 Å in DEQYOO01 [28].

**Figure 2:**
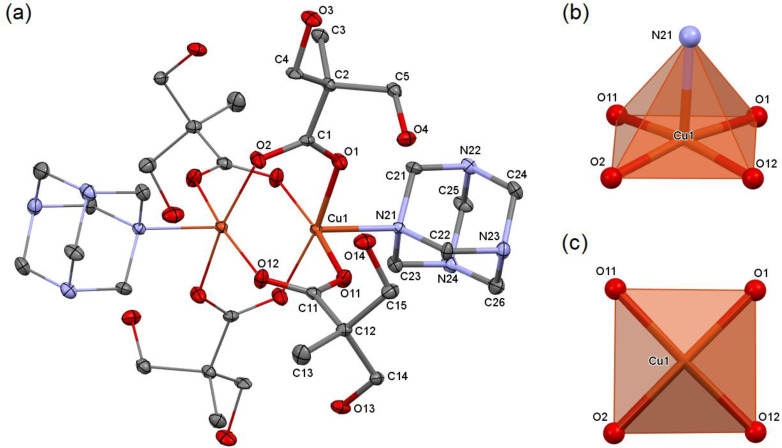
Molecular structure of 2, plotted with a 50% probability of displacement ellipsoids of nonhydrogen atoms (**a**). The equivalent atoms (without labels) were generated according to transformation: −*x* + 1, −*y* + 1, −*z* + 1. Hydrogen atoms were omitted for clarity. Coordination polyhedron of 2, general view (**b**), tetragonal base (**c**).

The synthesis in the 1:1 molar ratio results in the formation of a dinuclear compound [Cu_2_(dmp)_4_(hmta)_2_] (2). Four bidentate bridging (syn–syn) dmp anions and two monodentate hmta molecules create a paddle-wheel structure (Figure 2). The asymmetric unit contains half of the coordination moiety due to an inversion center between the copper atoms (special position *h* of the *P*-1 space group). The coordination polyhedron adopts the geometry of a tetragonal pyramid [29], whose apex is occupied by a hmta nitrogen atom (Figure 2). There is no asymmetricity of the coordination bonds formed by bridging dmp anions. All Cu–O bonds are of similar lengths (Table 1).

**Figure 3:**
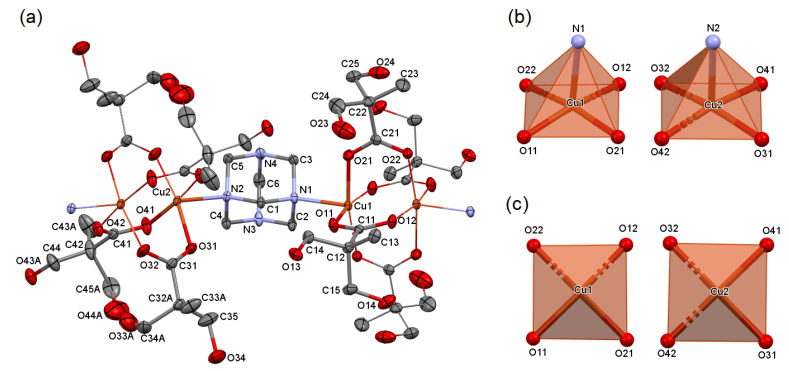
Molecular structure of 3, plotted with a 50% probability of displacement ellipsoids of nonhydrogen atoms (**a**). The equivalent atoms (without labels) were generated according to transformations: −*x*, −*y*, −*z* and −*x* + 1, −*y* + 1, −*z* + 1. Hydrogen atoms and parts with distorted dmp anions of lower contribution were omitted for clarity. Coordination polyhedra of 3, general view (**b**), tetragonal bases (**c**).

Both syntheses with a hmta deficiency i.e., in the molar ratio 2:1 and 3:1, led to the formation of the same compound [Cu_2_(dmp)_4_(hmta)]_n_ (3). It is a one-dimensional coordination polymer with a zigzag chain topology (Figure S2) [11]. Similar to 2, two copper cations are bridged by four dmp anions, resulting in a paddle-wheel moiety (Figure 3). The polymeric chains are formed because of the hmta molecules, which act as bidentate bridging ligands connecting the dinuclear copper entities. All chains are parallel to each other and they propagate along the [1 1 1] crystallographic direction. The asymmetric unit contains one hmta molecule and the halves of two paddle-wheel moieties due to the inversion centers between the copper atoms (special positions *a* and *h* of the *P*-1 space group). The analogical 1D coordination polymers of formula [Cu_2_(A)_4_(hmta)]_n_ (where A is a carboxylate anion) were reported with formate (CSD Refcode: RIMCAT [30]), acetate (BABDEN [31]), and 2,6-difluorobenzoate (VEMTEP [32]). Like 3, all mentioned polymers possess the zigzag topology of a single chain; however, they have completely different mutual arrangements of chains (Figure S2). This is a consequence of the different intermolecular interactions resulting from various structures of carboxylate anions. In formate and acetate compounds, the weak C—H•••O and C—H•••N hydrogen bonds stabilize the crystal structure of formate and acetate compounds, respectively. In the formate compound, hmta acts as a donor and formate as an acceptor of hydrogen bonds, whereas in the acetate compound the roles are reversed. Therefore, the polymeric chains are perpendicular in the former, and parallel in the latter. In 3 and 2,6-difluorobenzoate compounds, the distances between the polymeric chains are larger than in the formate and acetate analogs due to the ligand size. The mutual arrangement of chains in 3 is managed by O—H•••O and O—H•••N hydrogen bonds, while in the 2,6-difluorobenzoate compound, it is managed by π•••π and C—H•••π interactions.

The total valences of copper cations from dinuclear entities are 2.009 v.u. for 2, 1.970 v.u. and 2.020 v.u. for 3 (Table 1). It confirms that the Cu•••Cu interactions have a nonbinding character [33]. The quantum-mechanical calculations reveal an antiferromagnetic coupling between copper atoms in both compounds, which is typical for the paddle-wheel copper carboxylate system [34,35,36].

The O—H•••O and O—H•••N hydrogen bonds are the most important intermolecular interactions stabilizing the crystal structures of the studied compounds (Table 2). The hydroxyl groups of dmp anions and the water molecule (in 1) act as donors. The acceptors are oxygen atoms of the hydroxyl or carboxylate group of dmp anions and nitrogen atoms of hmta molecules. The hydrogen bonds in the studied compounds mainly form chain (C) and cyclic (R) motifs in the first level graph. The intramolecular hydrogen bonds between hydroxyl groups of dmp anions occur in the structures with paddle-wheel moieties (2 and 3). There are two equivalent S motifs per each dinuclear entity.

### 2.2. IR Spectroscopy Analysis

The spectral analyses were performed for all coordination compounds under the same experimental conditions to allow comparisons. Most of the vibrational modes appear to be strongly overlapped, and only the predominant contributions are indicated in Table 3.

The differences in the carboxylate and hmta ligand coordination mode are reflected in the position of some bands in the IR spectra (Figure S3). Owing to the strong asymmetricity of the coordination bonds formed by chelating dmp anions in 1, the positions of bands corresponding to the stretching vibrations (*ν*) of the carboxylate group are in a range that is characteristic of *ν* C=O and *ν* C-O. The separation of these two bands in 1 (Δ*ν* = 296 cm^−1^) is similar to that in undissociated dmpa (Δ*ν* = 288 cm^−1^) [37]. In the case of compounds 2 and 3, the positions of bands corresponding to carboxylate group vibrations are different than in 1. There is an observed decrease in the asymmetrical vibration frequency (*ν*_as_ COO) and an increase in the symmetrical vibration frequency (*ν*_a_ COO) in comparison, respectively, to *ν* C=O and *ν* C-O in pure acid, which is characteristic of the bidentate bridging mode. In addition, the vibrational frequencies of the OH group are similar for undissociated acid and carboxylate ligand. As far as hmta is considered, the splitting of the CN stretching frequencies (1240 and 1000 cm^−1^) indicates this ligand coordination mode [38]. In 3, these bands are split into well-defined and well-separated bands which is consistent with the bidentate bridging mode of hmta in this compound, whereas for 1 and 2 only very minor splitting is observed because hmta acts as a terminally bonded monodentate ligand. For all coordination compounds, the N–C–N bending vibrations appear as bands at ca. 670 cm^−1^ and 510 cm^−1^. The metal–ligand stretching vibrations have been attributed to the weak bands located in the low frequencies region.

### 2.3. UV-Vis Spectroscopy Analysis

Both calculated and experimental spectra of the studied compounds exhibit three distinguishable maxima. The calculated maxima are similar to the experimental ones (Figure S4). The most noticeable difference is seen in the case of 3. It must be outlined that the TD-DFT method works better for closed-shell systems [39]. Each studied compound possesses an open-shell coordination center. While 1 has only one coordination center, the complexity of the coordination centers for 2 and 3 is greater. Compound 2 is a dinuclear coordination compound in which copper centers can produce a different electronic spin-state system. Compound 3 is a coordination polymer in which the hmta molecule bridges dinuclear entities. These dinuclear entities can also produce a different electronic spin-state system. The process of wave function optimization led to electronic states with the lowest energies, for which, in the dinuclear entities of 2 and 3, the copper cations are coupled antiferromagnetically. In 3, however, the open-shell coordination centers are present in a more complex system. It also must be noted that only part of 3 was considered in the quantum-mechanical calculation. These factors might have affected the calculated spectrum of 3.

There are observed significant differences for the first two maxima when comparing these maxima in 1 with 2 and 3. In 1, those maxima are observed for considerably lower wavelengths, about 60–80 nm, than they are observed in 2 and 3 (Table 4, Figure S4). The same kind of difference is also observed for calculated spectra. For 3, however, those calculated maxima strongly shift towards larger wavelengths than observed in this compound’s experimental spectrum. All the above results are due to differences in the coordination environment of the copper cation. In 1, unlike in 2 and 3, a copper cation is coordinated by the nitrogen atoms of the hmta molecules located in the axial positions. Additionally, a water molecule coordinates this cation to an equatorial position. As a result, the copper cation becomes richer in electrons and adopts a geometrical pattern described as a distorted pentagonal bipyramid. More electron-filled orbitals of a copper cation are less involved in these particular transitions. Hence in 1, the first maximum is caused by ligand-to-ligand charge transfer (LLCT), which might be considered as coupled n→σ*, σ→σ*, and n→π* transitions (Table 4, Figures S5–S7). On the contrary, compounds 2 and 3 possess copper cations whose coordination patterns are described as tetragonal pyramids. In these compounds, there are dinuclear entities in which dmp ligands bridge copper cations. Copper cations in these entities are not as rich in electrons as they are in 1. Hence, both in 2 and 3, the first maximum is mainly caused by ligand-to-metal charge transfer (LMCT) and metal-to-metal charge transfer (MMCT) transitions. The second maximum in the spectrum of 1 involves complex molecular transitions, which seem to be mainly LMCT (Table 4, Figures S5–S7). This maximum in 2 and 3 seems to also be caused by LMCT, but the particular molecular orbitals involved in the transitions are simpler in those compounds. In all compounds, donor transition orbitals are mainly localized around copper coordination centers (Figures S5–S7). However, in 1, unlike 2 and 3, those orbitals also engage hmta ligands. The third maximum is mainly due to transitions involving sigma and lone pair orbitals of dmp ligands and d orbitals of copper cations (Table 4, Figures S5–S7). As was the case with the second maximum, the donor transition orbitals are mainly localized around copper coordination centers.

### 2.4. Thermal Analysis

The thermal analyses of the studied compounds showed that their decomposition pathways are similar (Figures S8–S10). The thermal decomposition of 1 starts from a dehydration process (Table 5, Figure S11). The release of water molecules occurs at higher temperatures (above 100 °C) due to their binding to copper cations. For all studied compounds, the decomposition of dmp and hmta co-occurs at a similar temperature range (Table 5). For 2 and 3, it is possible to distinguish two substages within this process. The mass spectra registered for this stage contain typical signals for combustion products like C^+^, H_2_O^+^, NO^+^, and CO_2_^+^, as well as signal sets of fragmentation ions of hmta, e.g., HCN^+^, C_2_H_4_N^+^, C_2_H_4_N_2_^+^, C_4_H_8_N_2_^+^ C_5_H_10_N_2_^+^, and dmp, e.g., CH_3_^+^, OH^+^, CH_2_OH^+^ (Figure S12). The disintegration of ligands is not a complete process and thus, the experimental mass losses are lower than theoretical ones. A random amount of pyrolytic soot is formed in all studied cases, which is a common phenomenon for hmta coordination compounds [20]. The combustion of such residue is a strongly exothermic process (Figures S8–S10). The main products registered in the mass spectra from this stage are C^+^, NH_3_^+^/OH^+^, H_2_O^+^, HCO^+^, NO^+^, and CO_2_^+^ (Figure S13). The final product in all studied cases is a mixture of copper(II) oxide (tenorite, a = 4.684 Å, b = 3.425 Å, c = 5.129 Å, β = 99.47°, Z = 4, space group *C*2/*c*, no. 15) and copper(I) oxide (cuprite, a = 4.258 Å, Z = 2, space group *Pn*—3*m*, no. 224), which was confirmed by X-ray powder measurements (Figure S14). The dominant product is CuO, whose content in the final mixture is 78%, 85%, and 82%, respectively for 1–3. The amount of oxides remaining after decomposition is lower than expected for all studied compounds. The most probable explanation for this is that the random part of the copper content is removed during ligand disintegration. It is especially exemplified for 3, for which the experimental mass loss at this stage agrees with the theoretical loss but despite that the pyrolytic soot is formed. Moreover, there are no signals of m/z above 50 in the mass spectra from the pyrolytic soot combustion, which indicates that copper is not lost in the last decomposition stage.

## 3. Materials and Methods

### 3.1. Synthesis of Copper Coordination Compounds

Copper(II) 2,2-bis(hydroxymethyl)propionate was synthesized via the suspension of copper(II) carbonate hydroxide Cu_2_(OH)_2_CO_3_ (0.077 mol, 17.067 g) in a solution of 2,2-bis(hydroxymethyl)propionic acid (0.28 mol, 37.569 g in 1 dm^3^ of water). The mixture was heated at 100 °C under reflux for 6 h and then it was filtered to remove the unreacted excess of copper(II) carbonate hydroxide. The concentration of copper(II) 2,2-bis(hydroxymethyl)propionate in the final solution was determined via edta titration of copper(II) in the presence of 1-(2-pyridylazo)-2-naphthol as an analytical indicator [40], and it was 0.134 mol/dm^3^ (yield in relation to carboxylic acid, which was used in deficiency, was 96%). This solution was used for four syntheses with hmta, which were carried out in different copper:hmta molar ratios, i.e., 1:2, 1:1, 2:1, 3:1. The appropriate volumes of copper(II) 2,2-bis(hydroxymethyl)propionate solution containing, respectively, 0.01 mol, 0.01 mol, 0.02 mol, and 0.03 mol of copper salt were mixed with the solid samples of hmta (0.02 mol, 0.01 mol, 0.01 mol, and 0.01 mol, respectively). These reaction solutions were stirred with magnetic stirrers for 1 h and then they were left to crystallize at room temperature. Single crystals of different shades of blue were formed after 10–12 weeks. The copper(II) 2,2-bis(hydroxymethyl)propionate was not isolated from the solution after its synthesis, because it would have to be dissolved in water one more time before reaction with hmta.

### 3.2. Crystal Structure Determination

X-ray diffraction data of 1–3 were collected at 100.0(1) K, on a Rigaku Synergy Dualflex automatic diffractometer (Rigaku Corporation, Tokyo, Japan) equipped with a Pilatus 300 K detector and micro-focus sealed PhotonJet X-ray tube generated monochromatic Cu*K_α_* radiation (1.54184 Å), with shutterless ω scan mode. Lorentz, polarization, and empirical absorption (using spherical harmonics, implemented in the SCALE3 ABSPACK scaling algorithm) corrections were applied during the data reduction. The structures were solved with a dual-space algorithm (SHELXT [41]). All non-hydrogen atoms were refined anisotropically using a full-matrix, least-squares technique on *F*^2^ (SHELXL [42]). All hydrogen atoms were refined using the “riding” model. Isotropic displacement factors of hydrogen atoms were equal to 1.2 times the value of an equivalent displacement factor of parent methine carbon atoms, and 1.5 times the value of parent hydroxyl oxygen, water oxygen, and methyl carbon atoms. Two carboxylate anions in the structure of compound 3 are disordered over two positions with 0.66:0.33 and 0.54:0.46 participation of domains, respectively, for carboxylates containing C31 and C41 carbon atoms (Figure S15). The solvent mask function was used in the refinement of 3 due to disordered water molecules (impossible to refine) in an outer coordination sphere. It revealed that there is ca. one water molecule per unit cell. Structural visualizations were made using the Mercury 2020.2.0 software package (Cambridge Crystallography Data Centre, Cambridge, UK) [43]. Details concerning crystal data and refinement are given in Table S1.

### 3.3. Physicochemical Measurements

The FT-IR spectra were recorded on the Jasco FT/IR 6200 spectrophotometer (JASCO, Easton, MD, USA), in the form of KBr pellets, in the spectral range 4000–400 cm^−1^. The UV-Vis diffuse reflectance spectra were recorded on a Jasco V-660 spectrometer (Jasco, Easton, MD, USA), in the spectral range 190–800 nm, using spectralon [44] as a standard with 100% reflectance. The thermal decompositions were carried out with the Netzsch STA 449 F1 Jupiter thermoanalyzer (Netzsch-Geratebau GmbH, Selb, Germany) coupled with the Netzsch Aeolos Quadro QMS 403 mass spectrometer (Netzsch-Geratebau GmbH, Selb, Germany). Samples were heated in corundum crucibles, in the temperature range 35–1000 °C, with the heating rate of 10 °C/min in synthetic air (80% N_2_, 20% O_2_). The XRPD patterns were recorded in a reflection mode on the XPert PRO MPD diffractometer (Malvern Panalytical Ltd., Royston, UK) equipped with Cu*Kα*1 radiation, a Bragg–Brentano PW 3050/65 high-resolution goniometer, and PW 3011/20 proportional point detector.

### 3.4. Quantum-Mechanical Calculations

The excited states of 1–3 have been calculated for X-ray determined coordinates using the TD-DFT method. Input structural models were prepared with the Mercury 2020.2.0 software package [43]. In the case of each compound, positions of hydrogen atoms have been normalized by moving them along the covalent bond vector (X→H) to the X-H distance equal to the average neutron diffraction value. For 1 and 2 as an input, a coordination unit of a respective compound was used. For 3, the input was constructed to cover its two dinuclear subunits bridged with a hmta ligand. The adjacent hmta ligands were also left. All calculations were performed using Gaussian09 rev. E.01 (Gaussian Inc., Wallingford, CT, USA) [45] with functional B3LYP [46] and the SDD basis set—Dunning/Huzinaga full double zeta basis set (up to argon) and Stuttgart/Dresden ECPs (for heavier elements than argon [47]). As the compounds contain copper cations as coordination centers, in each case, the wave functions were subjected to the optimization process. The resulted electronic states were then used in the TD-DFT calculation. The number of calculated transitions was set to 40. The calculated excited states’ assignment to the observed experimental maxima was based on a comparison of excitation energies and the oscillator strengths/intensities of the corresponding maxima. The analysis of the character of respective orbital excitations was based on orbital contour plots.

## 4. Conclusions

The stoichiometry of reagents plays a key role in the formation of coordination compounds containing copper(II) 2,2-bis(hydroxymethyl)propionate and hexamethylenetetramine. The retainment of the Cu(dmp)_2_:hmta ratio within the solid products was possible for several reasons, i.e., the propensity of copper to create multinuclear compounds, the ability of hmta to form a diverse number of coordination bonds, and the tendency of dmp to coordinate via the carboxylate group whereby free hydroxyl groups can stabilize the structure through hydrogen bonding. The structural diversity of the obtained compounds results from the ligands’ ability to adopt multiple coordination modes, i.e., bidentate chelating and bidentate bridging by dmp, and monodentate and bidentate bridging by hmta. The structural differences reflect the spectral and thermal properties. The location and splitting of the bands attributed to the stretching vibration of the carboxylate group and C-N bonds correspond to the coordination modes of the ligands. The bidentate bridging mode of dmp in 2 and 3 causes a bathochromic shift of the absorption maxima and changes their character compared to the chelating dmp in 1. This work provides valuable knowledge on designing new coordination compounds for desired structure and properties.

## Figures and Tables

**Table 1:** Structural data of the coordination polyhedra in the studied compounds.

i—j	d_ij_ (Å)	*ν*_ij_ (v.u.)	i—j—k	α_ijk_ (°)	i—j—k	α_ijk_ (°)
compound 1						
Cu1—O1	1.9654 (13)	0.432	O1—Cu1—O2	47.90 (5)	O11—Cu1—O12	50.38 (5)
Cu1—O2	2.9953 (15)	0.027	O1—Cu1—O11	169.04 (6)	O11—Cu1—O41	94.59 (5)
Cu1—O11	1.9684 (13)	0.429	O1—Cu1—O12	118.70 (5)	O11—Cu1—N21	90.41 (5)
Cu1—O12	2.8616 (16)	0.038	O1—Cu1—O41	96.25 (5)	O11—Cu1—N31	90.03 (5)
Cu1—O41	2.1931 (13)	0.234	O1—Cu1—N21	90.87 (5)	O12—Cu1—O41	144.86 (5)
Cu1—N21	2.1154 (14)	0.337	O1—Cu1—N31	89.44 (5)	O12—Cu1—N21	91.55 (5)
Cu1—N31	2.1272 (15)	0.326	O2—Cu1—O11	121.35 (5)	O12—Cu1—N31	91.72 (5)
			O2—Cu1—O12	71.14 (4)	O41—Cu1—N21	91.36 (5)
			O2—Cu1—O41	144.00 (4)	O41—Cu1—N31	84.73 (5)
			O2—Cu1—N21	86.66 (5)	N21—Cu1—N31	176.09 (6)
			O2—Cu1—N31	96.42 (5)		
compound 2						
Cu1—O1	1.9652 (12)	0.431	O1—Cu1—O2(i)	169.67 (5)	O2(i)—Cu1—O12 (i)	89.22 (6)
Cu1—O2(i)	1.9752 (13)	0.424	O1—Cu1—O11	88.12 (6)	O2(i)—Cu1—N21	99.10 (5)
Cu1—O11	1.9562 (13)	0.440	O1—Cu1—O12(i)	89.42 (6)	O11—Cu1—O12(i)	169.53 (5)
Cu1—O12(i)	1.9631 (13)	0.435	O1—Cu1—N21	91.21 (5)	O11—Cu1—N21	95.78 (5)
Cu1—N21	2.1839 (14)	0.280	O2(i)—Cu1—O11	91.37 (6)	O12(i)—Cu1—N21	94.45 (5)
Cu1•••Cu1(i)	2.5927 (5)					
compound 3						
Cu1—O11	1.9624 (16)	0.436	O11—Cu1—O12(ii)	168.86 (7)	O12(ii)—Cu1—O22(ii)	88.06 (7)
Cu1—O12(ii)	1.9761 (16)	0.420	O11—Cu1—O21	90.17 (7)	O12(ii)—Cu1—N1	88.76 (7)
Cu1—O21	1.9688 (16)	0.428	O11—Cu1—O22(ii)	88.67 (7)	O21—Cu1—O22(ii)	169.29 (7)
Cu1—O22(ii)	1.9691 (16)	0.428	O11—Cu1—N1	102.21 (7)	O21—Cu1—N1	93.73 (7)
Cu1—N1	2.2135 (18)	0.259	O12(ii)—Cu1—O21	91.06 (7)	O22(ii)—Cu1—N1	96.92 (7)
Cu1•••Cu1(ii)	2.6027 (6)					
Cu2—O31	1.9503 (17)	0.450	O31—Cu1—O32(i)	169.32 (7)	O32(i)—Cu1—O42(i)	89.87 (8)
Cu2—O32(i)	1.9595 (17)	0.439	O31—Cu1—O41	89.25 (9)	O32(i)—Cu1—N2	96.71 (7)
Cu2—O41	1.9626 (17)	0.435	O31—Cu1—O42(i)	90.22 (8)	O41—Cu1—O42(i)	169.54 (7)
Cu2—O42(i)	1.9717 (16)	0.425	O31—Cu1—N2	93.95 (7)	O41—Cu1—N2	96.72 (7)
Cu2—N2	2.1973 (18)	0.270	O32(i)—Cu1—O41	88.73 (9)	O42(i)—Cu1—N2	93.74 (7)
Cu2•••Cu2(i)	2.5897 (6)					

The bond valences were calculated as *ν*_ij_ = exp*[(R*_ij_*-d*_ij_*)/b*] [22,23], where *R*_ij_ is the bond-valence parameter for i–j bond (*R*_Cu-N_ = 1.713; *R*_Cu-O_ = 1.655 Å [24]) and *b* is the constant equalled 0.37 Å [25]. Symmetry transformations used to generate equivalent atoms: (i) −*x* + 1, −*y* + 1, −*z* + 1; (ii) −*x*, −*y*, −*z*.

**Table 2:** Hydrogen bonds in the studied compounds.

D—H•••A	d(D—H) (Å)	d(H•••A) (Å)	d(D•••A) (Å)	<(DHA) (°)	Graph-Set
compound 1					
O3—H3•••N22(i)	0.84	2.17	2.942 (2)	153	C (10)
O4—H4•••N24(ii)	0.84	2.01	2.840 (2)	170	R_2_^2^ (20)
O13—H13•••N33(iii)	0.84	2.01	2.850 (2)	177	R_2_^2^ (20)
O14—H14•••N34(iv)	0.84	2.16	2.936 (3)	154	C (10)
O41—H41O•••O12(v)	0.86	1.83	2.636 (2)	157	C (6)
O41—H41P•••O2(v)	0.87	1.82	2.6373 (19)	155	C (6)
compound 2					
O3—H3•••O13(vi)	0.84	2.05	2.867 (3)	164	C (12)
O4—H4•••O14(vii)	0.84	1.90	2.737 (3)	179	C (12)
O13—H13•••N22(viii)	0.84	1.92	2.759 (3)	179	C (10)
O14—H14•••O4	0.84	1.96	2.770 (3)	162	S (12)
compound 3					
O13—H13•••N3(v)	0.84	1.97	2.791 (3)	166	C (10)
O14—H14•••O24(ix)	0.84	1.87	2.711 (4)	176	S (12)
O23—H23•••O34(x)	0.84	2.09	2.786 (4)	140	C (16)
O24—H24•••O13(vi)	0.84	1.89	2.727 (3)	172	C (12)
O33A—H33D•••O43A(xi)	0.84	2.36	2.769 (5)	111	R_2_^2^ (32)
O34—H34•••O14(xii)	0.84	1.94	2.765 (4)	166	R_2_^2^ (32)
O43A—H44D•••O23(xiii)	0.84	2.03	2.855 (4)	169	S (16)

Symmetry transformations used to generate equivalent atoms: (i) x + 1, y + 1, z; (ii) −x + 1, −y, −z + 1; (iii) −x + 1, −y + 1, −z; (iv) x, y + 1, z; (v) x−1, y, z; (vi) x, y−1, z; (vii) −x + 1, −y, −z + 2; (viii) x + 1, y, z; (ix) −x, −y, −z; (x) x−1, y−1, z; (xi) −x + 2, −y + 2, −z + 1; (xii) −x, −y + 1, −z; (xiii) −x + 1, −y + 1, −z + 1.

**Table 3:** Vibrational frequencies (cm^−1^) and their assignments for the studied compounds.

1	2	3	Dmpa [37]	Hmta [38]	Assignment
3336	3407	3436	3368		*ν* OH
3228			3230		*ν* OH
		2973		2966	*ν* CH
2944	2941			2955	*ν* CH
2925				2919	*ν* CH
2873	2879	2885		2872	*ν* CH
1703			1691		*ν* C=O, *δ* OH_(water)_
		1701			*δ* OH_(water)_
	1627	1621			*ν*_as_ COO, *δ* OH_(water)_
1599		1570			*ν* CN
1460	1466	1467	1456	1456	*δ* CH
	1423	1421			*ν*_a_ COO
1407			1403		*ν* C-O
1374	1367	1383		1370	*δ* CH
1323	1298	1294	1309		*δ* OH_(hydroxyl)_
1237	1236	1246	1236	1240	*ν* CO_(hydroxyl),_ *δ* CH, *ν* CN
		1232			*ν* CN
1056			1052		*δ* OH_(hydroxyl)__,_ *ν* CC
		1026			*ν* CN
1004	1001	1000		1007	*ν* CN
925			939		*δ* OH_(hydroxyl)_
888	891	891	870		*ν* CC
812	818			812	*ν* CN
776	782	787			*σ* COO
671	677	674		672	*δ* NCN
512	514	506		512	*δ* NCN
434	444	420			*ν* Cu–N
417	420	406			*ν* Cu–O

Vibrations symbols: *ν*—stretching, *δ*—bending, σ—scissoring, s—symmetric, as—asymmetric.

**Table 4:** The most important electronic transitions. H letter indicates HOMO, L—LUMO, α—α orbitals, β—β orbitals, and +/−(number) represents subsequent orbitals above HOMO and LUMO, respectively.

Experimental λ (nm)			Theoretical λ (nm)			Orbitals Involved in Electronic Transitions	Character of Transition
1	2	3	1	2	3		
194	263	275	217			αH − 2→αL + 1 αH − 1→αL + 1	d(Cu)/n&σ(hmta)→σ*(-OH of dmp)/σ*(H_2_O)
			219			αH→αL + 1 βH − 1→βL + 2	
			235			αH − 5→αL + 4 βH − 4→βL + 5	n(dmp)→σ*&π*(dmp) n&σ(dmp)→σ*&π *(dmp)
				258		αH − 18→αL βH − 18→βL	d(Cu)/n&σ(dmp)→d(Cu)/σ*(dmp-Cu)
				262		αH − 16→αL βH − 16→βL	
				279		αH − 13→αL βH − 13→βL	
					381	βH − 9→βL βH − 10→βL	d(Cu)/n&σ(dmp)/n&σ(hmta)→d(Cu)/σ*(dmp-Cu) d(Cu)/n&σ(dmp)/n(hmta)→d(Cu)/σ*(dmp-Cu)
					383	αH − 9→αL	d(Cu)/n&σ(dmp)→d(Cu)/σ*(dmp-Cu)
301	382	381	300			βH − 8→βL	d(Cu)/n&σ(dmp)/n&σ(hmta)→d(Cu)/σ*(dmp-Cu)/σ *(hmta-Cu)
			308			βH − 9→βL βH − 10→βL	
			350			βH − 6→βL βH − 8→βL	d(Cu)/n(-COO of dmp)n&σ(hmta)→d(Cu)/σ*(dmp-Cu)/σ*(hmta-Cu) d(Cu)/n&σ(dmp)/n&σ(hmta)→d(Cu)/σ*(dmp-Cu)/σ*(hmta-Cu)
				361		αH − 6→αL βH − 6→βL	d(Cu)/n&σ(dmp)→d(Cu)/σ*(dmp-Cu)
					461	αH − 4→αL	n&σ(hmta)→d(Cu)/σ*(dmp-Cu)
					471	αH − 3→αL	
					483	βH→βL	
			621			βH − 19→βL	d(Cu)/σ(dmp)→d(Cu)/σ*(dmp-Cu)/σ*(hmta-Cu)
733	688	686		604		αH − 21→αL βH − 21→βL	d(Cu)/n(dmp-Cu)→d(Cu)/σ*(dmp-Cu)
				607		αH − 33→αL βH − 33→βL	d(Cu)/n&σ(dmp)→d(Cu)/σ*(dmp-Cu)
				614		αH − 34→αL βH − 34→βL	
					606	βH − 63→βL + 1	d(Cu)/n&σ(dmp)→d(Cu)/σ*(dmp-Cu)
					632	βH − 25→βL βH − 67→βL	d(Cu)/n&σ(dmp)→d(Cu)/σ*(dmp-Cu) d(Cu)/n&σ(dmp)/σ(hmta)→d(Cu)/σ*(dmp-Cu)

Used abbreviations: d—d orbital, n—non-bonding orbital, σ—σ orbital, π—π orbital, n&σ—n and σ orbital, σ&π—σ and π orbital, *—an antibonding orbital, dmp—2,2-bis(hydroxymethyl)propionate anion, hmta—hexamethylenetetramine, -OH—hydroxyl group, -COO—carboxylate group, dmp-Cu—a part of 2,2-bis(hydroxymethyl)propionate anion close to a copper cation.

**Table 5:** Temperature ranges and mass losses (experimental/theoretical) of thermal decomposition stages.

Process/Final Product	1	2	3
Dehydration	105–158 °C 1.9%/2.9%	-	-
Simultaneous disintegration of dmp and hmta	158–315 °C 67.6%/84.5%	Substage 1 154–232 °C 27.6%/−	Substage 1 156–237 °C 26.3%/−
		Substage 2 232–305 °C 39.8%/−	Substage 2 237–316 °C 54.7%/−
		Totally 67.4%/83.1%	Totally 81.0%/80.1%
Combustion of pyrolitic soot	315–565 °C 25.6%/−	305–485 °C 18.4%/−	316–495 °C 10.1%/−
CuO + Cu_2_O	565 °C 4.9%/12.7% *	485 °C 14.2%/16.9% *	495 °C 8.9%/19.9% *

* theoretical percentage of the final product was calculated with an assumption of pure CuO formation.

## Data Availability

The data presented in this study are available on request from the corresponding author. The CIF files containing full crystallographic data for compounds 1–3 (CCDC 2081741–2081743) can be obtained free of charge via http://www.ccdc.cam.ac.uk/conts/retrieving.html, accessed on 2 June 2021 (or from the CCDC, 12 Union Road, Cambridge CB2 1EZ, UK; Fax: +44-1223-336033; E-mail: deposit@ccdc.cam.ac.uk).

## Supplementary Materials

The following are available online, Figure S1: (a) Overlapping of molecular natural bond orbitals (NBOs) in Cu1/O2/O12 plane of 1. (b) The calculated charge density in Cu1/O2/O12 plane of 1, Figure S2: The arrangement of polymeric chains in the crystal structure of 3 and analogs compounds of formula [Cu2(A)4(hmta)]n reported in the literature., Figure S3: FT-IR spectra of 1 (red), 2 (green), and 3 (blue), Figure S4: Experimental and calculated UV-Vis spectra of 1-3, Figure S5: Calculated molecular orbitals of the compound 1: α (a) and β (b), Figure S6: Calculated molecular orbitals of the compound 2: α (a) and β (b), Figure S7: Calculated molecular orbitals of the compound 3: α (a) and β (b), Figure S8: TG, DTA, and DTG curves for 1, Figure S9: TG, DTA, and DTG curves for 2, Figure S10: TG, DTA, and DTG curves for 3, Figure S11: Mass spectrum of volatile products registered at 135 °C during thermal decomposition of 1, Figure S12: Mass spectrum of volatile products registered at 250 °C during thermal decomposition of 1, Figure S13: Mass spectrum of volatile products registered at 470 °C during thermal decomposition of 1, Figure S14: The XRPD pattern of the final product obtained after thermal decomposition of 1 and reference patterns of tenorite PDF-2 code 00-005-0661 and cuprite PDF-2 code 01-077-0199, Figure S15: Disorder models of two dmp anions of 3, Table S1: Crystal data and structure refinement details for the studied compounds.

## Sample Availability

Samples of the compounds are available from the authors.
