# Supplementary material for: Copper(II) 2,2-Bis(Hydroxymethyl)Propionate Coordination Compounds with Hexamethylenetetramine: From Mononuclear Complex to One-Dimensional Coordination Polymer
Source: Molecules. 2021 Jun 2;26(11):3358. doi: 10.3390/molecules26113358 (PMC8199667; doi:10.3390/molecules26113358)
Supplement: Supplementary file 1 [file molecules-26-03358-s001.zip › molecules-1238753-supplementary.pdf]

molecules

Supplementary Materials

Copper(II) 2,2-Bis(hydroxymethyl)propionate Coordination Compounds with Hexamethylenetetramine: from Mononuclear Complex to One-dimensional Coordination Polymer

Sadaf Rauf, Agata Trzesowska-Kruszynska, Tomasz Sierański, Marcin Świątkowski

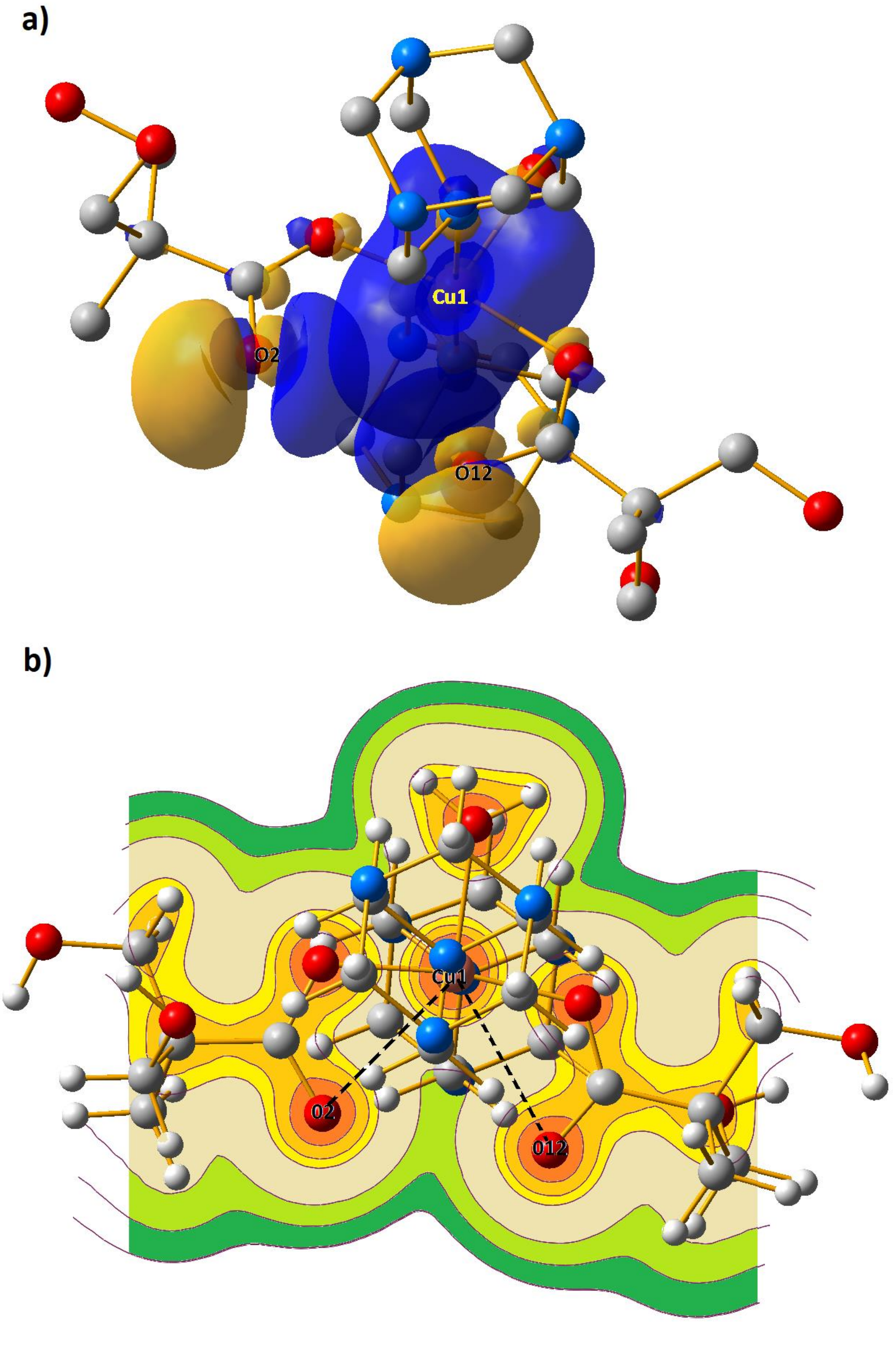

Figure S1. (a) Overlapping of molecular natural bond orbitals (NBOs) in Cu1/O2/O12 plane of **1**. The NBOs were drawn with the isovalue equal to 0.05 au. Hydrogen atoms were removed to enhance clarity. (b) The calculated charge density in Cu1/O2/O12 plane of **1**. All calculations were performed at B3LYP/SDD level of theory utilizing Gaussian09 rev. E.01.

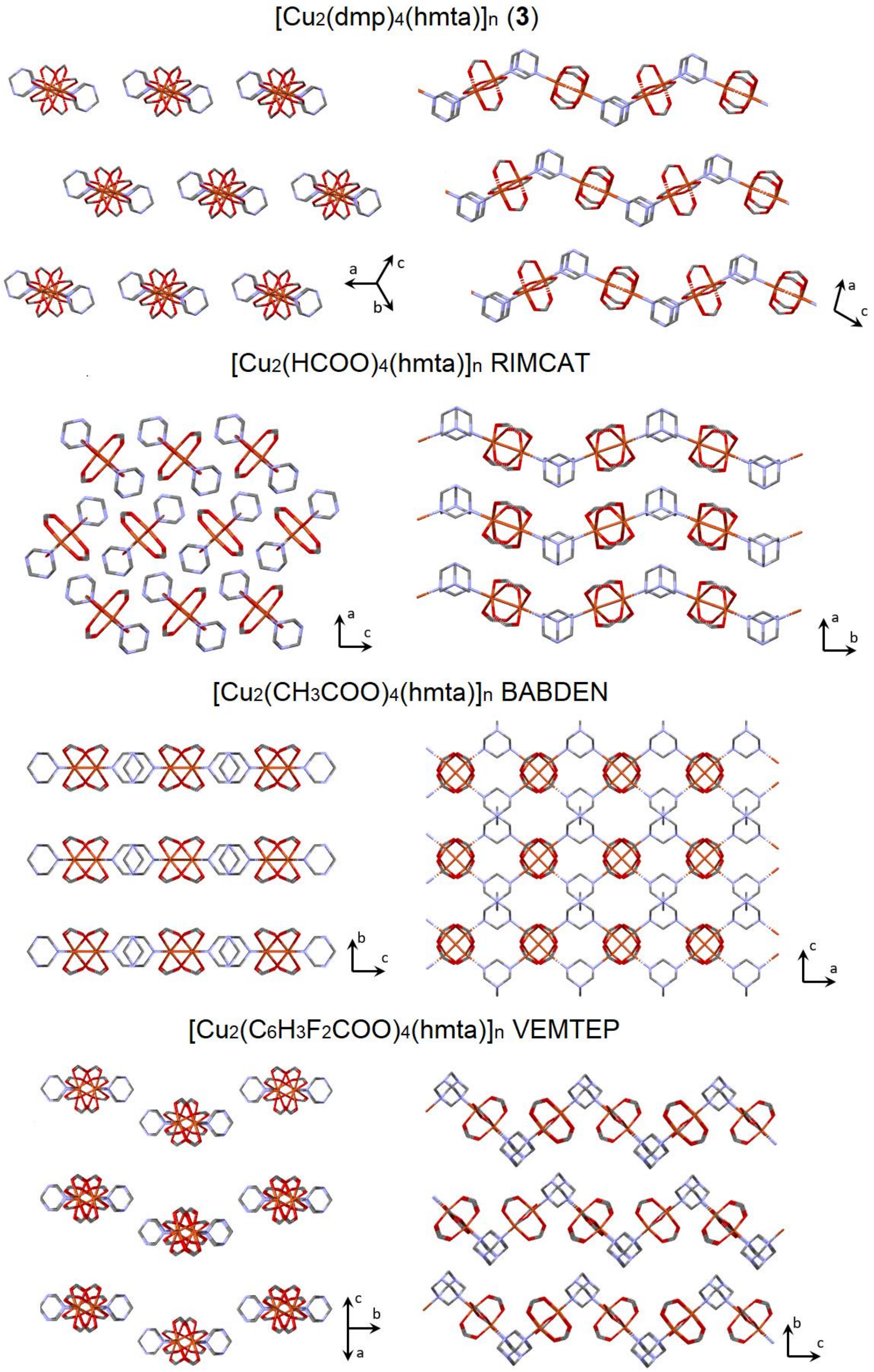

Figure S2. The arrangement of polymeric chains in the crystal structure of **3** and analogous compounds of formula $[Cu_2(A)_4(hmta)]_n$ reported in the literature. Hydrogen atoms and parts of carboxylate anions excluding carboxylate groups were omitted for clarity.

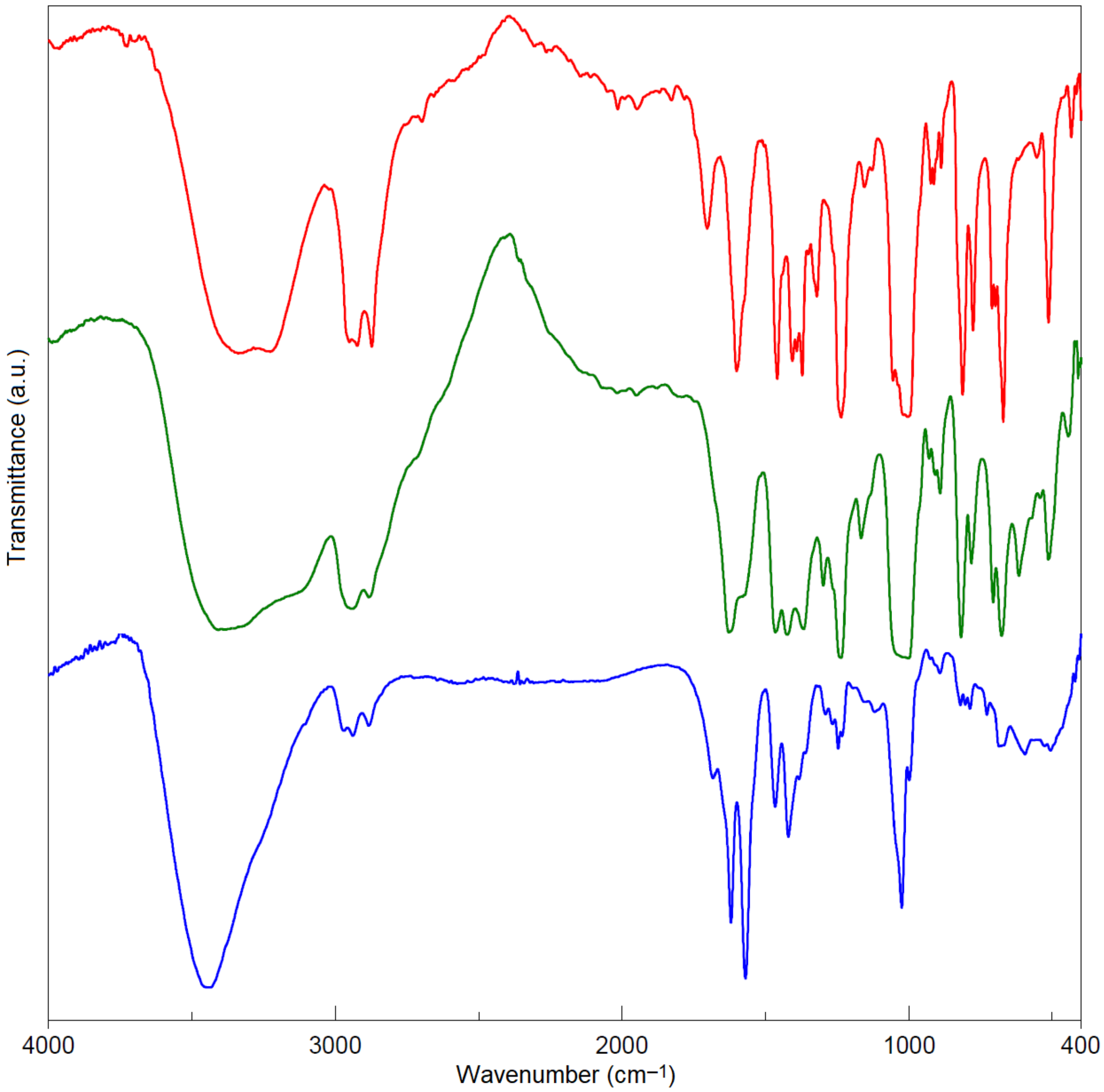

Figure S3. FT-IR spectra of **1** (red), **2** (green), and **3** (blue).

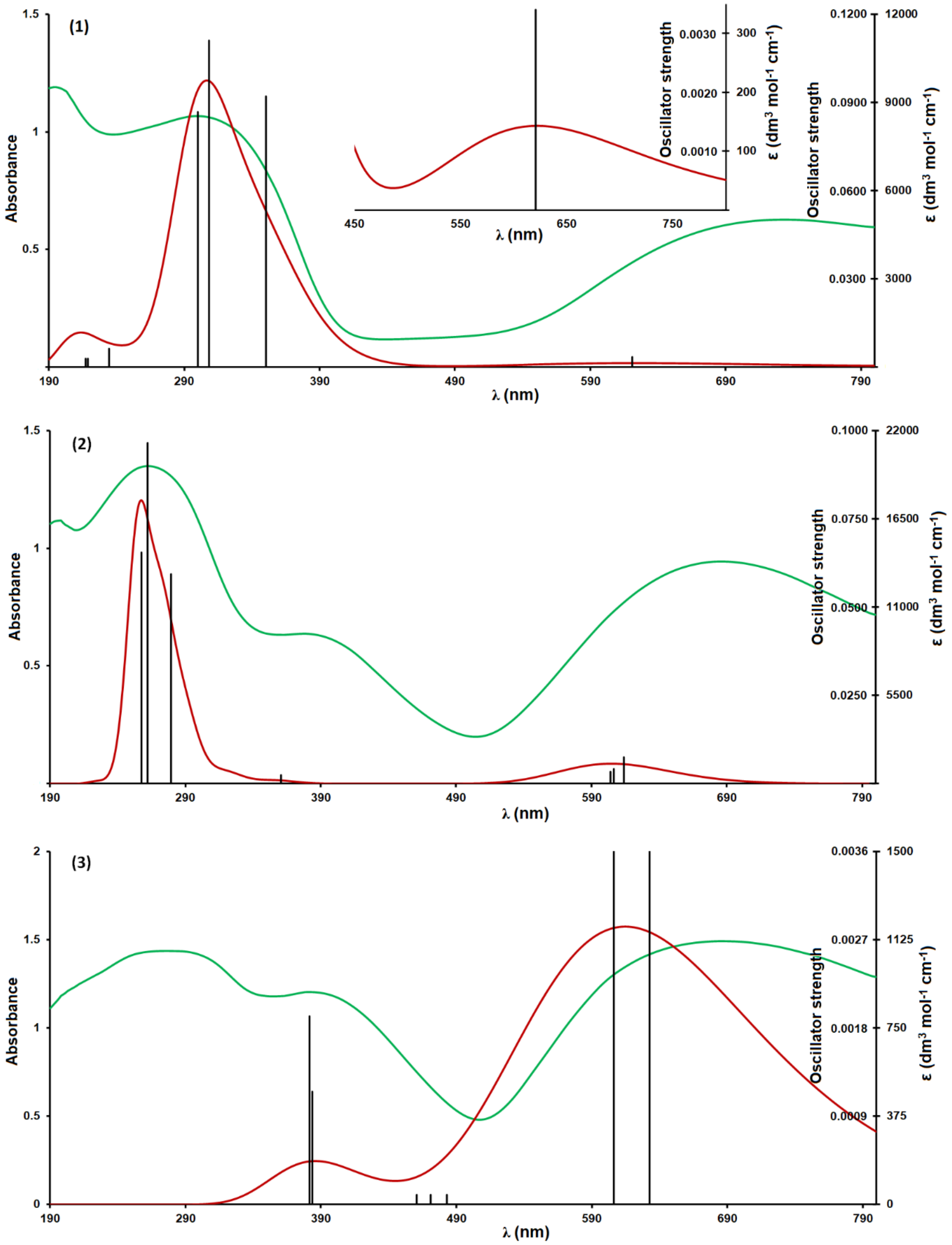

Figure S4. Experimental (green) and calculated (red) UV-Vis spectra of **1**-**3** The most important oscillator strengths are shown as vertical black lines.

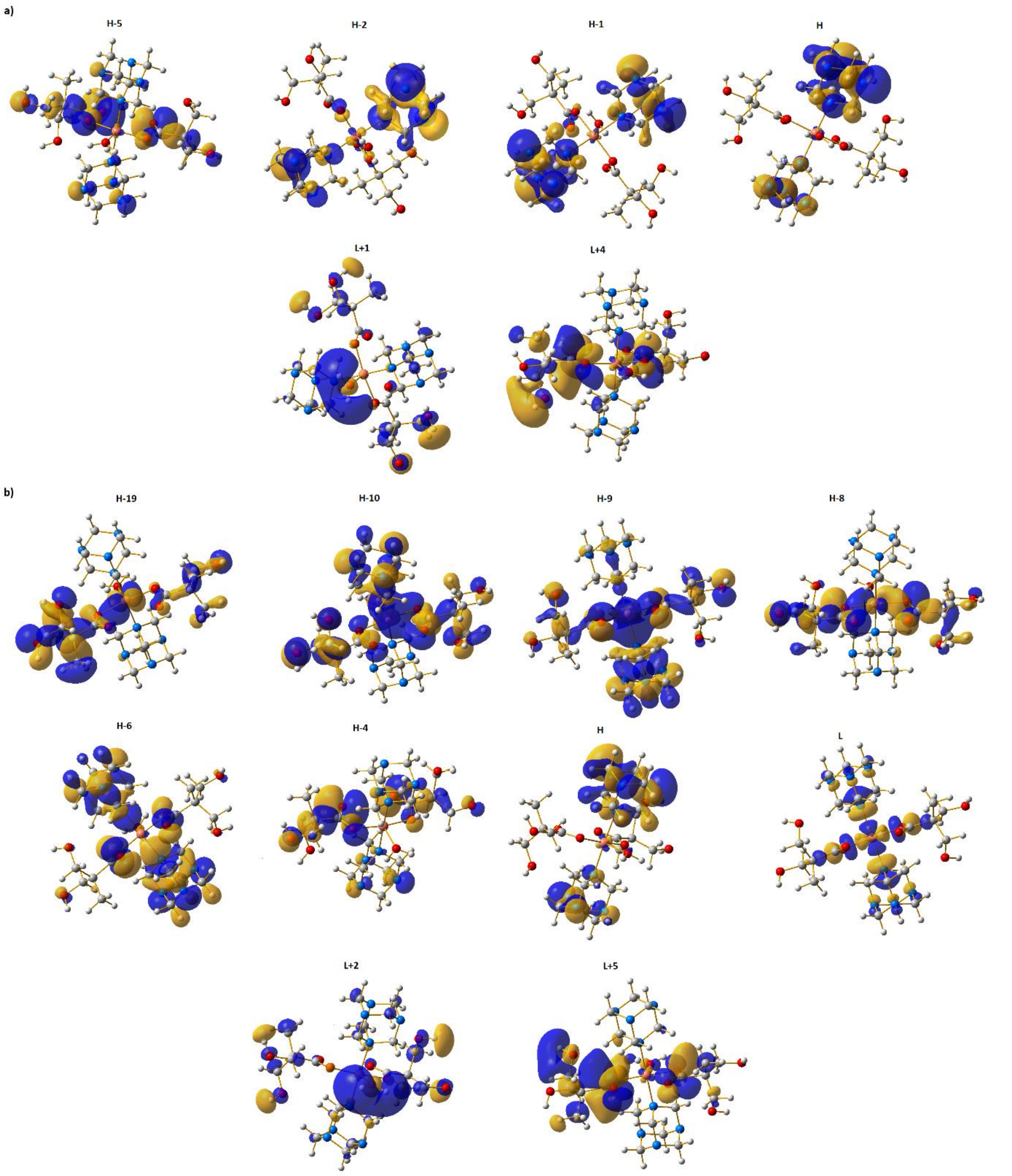

Figure S5. Calculated molecular orbitals of the compound **1**: α (a) and β (b). H letter indicates HOMO, L - LUMO, and +/-(number) represents subsequent orbitals below HOMO and above LUMO, respectively.

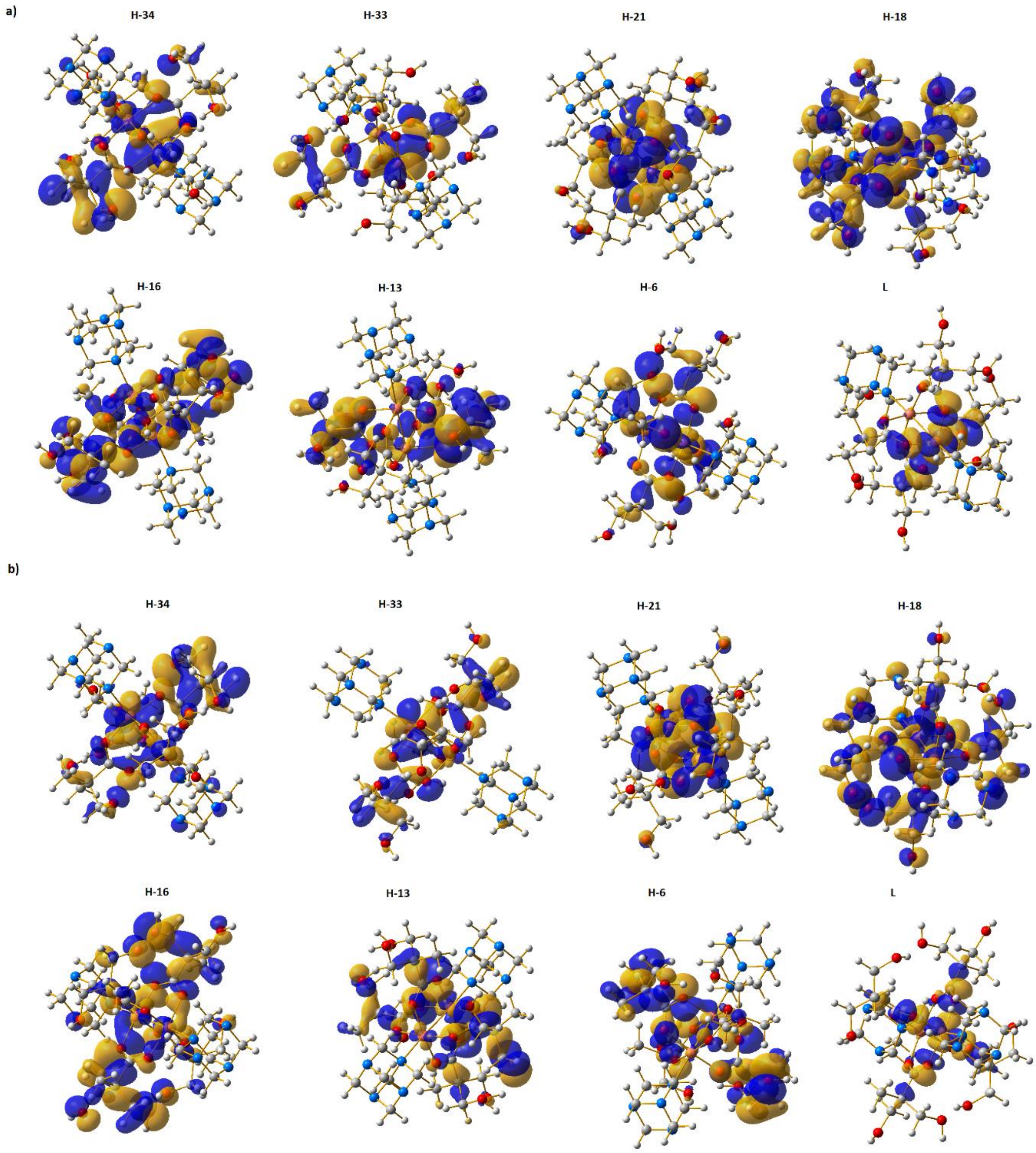

Figure S6. Calculated molecular orbitals of the compound **2**: α (a) and β (b). H letter indicates HOMO, L - LUMO, and +/-(number) represents subsequent orbitals below HOMO and above LUMO, respectively.

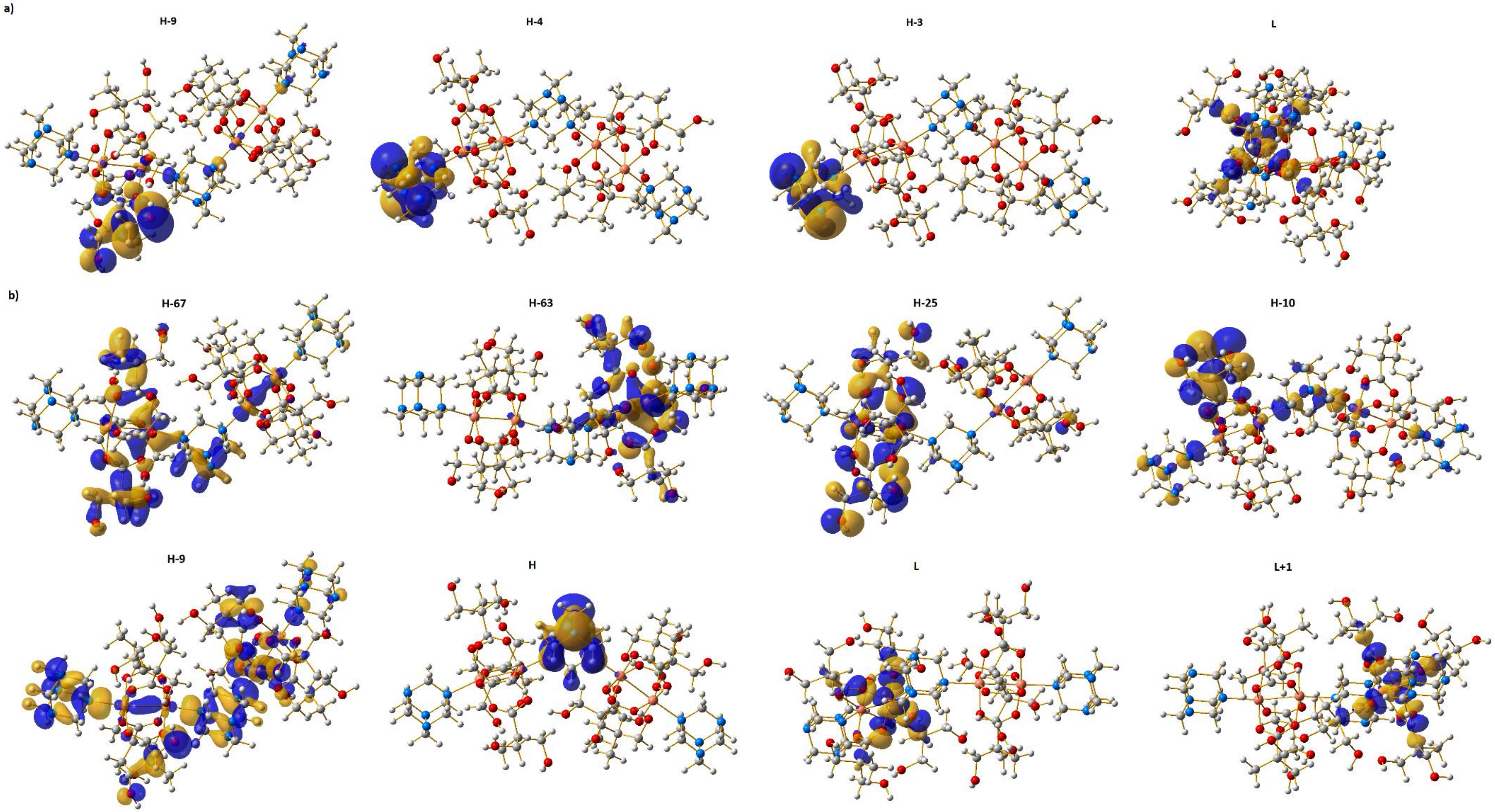

Figure S7. Calculated molecular orbitals of compound **3**: α (a) and β (b). H letter indicates HOMO, L - LUMO, and +/-(number) represents subsequent orbitals below HOMO and above LUMO, respectively.

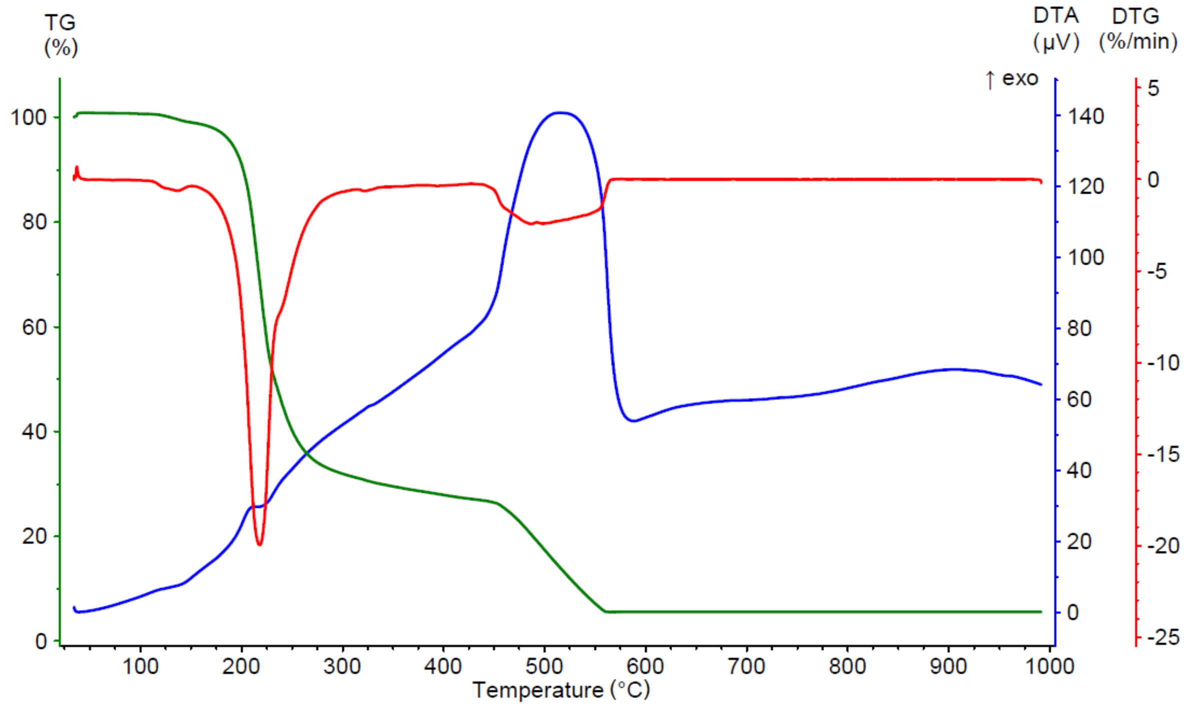

Figure S8. TG, DTA, and DTG curves for **1**.

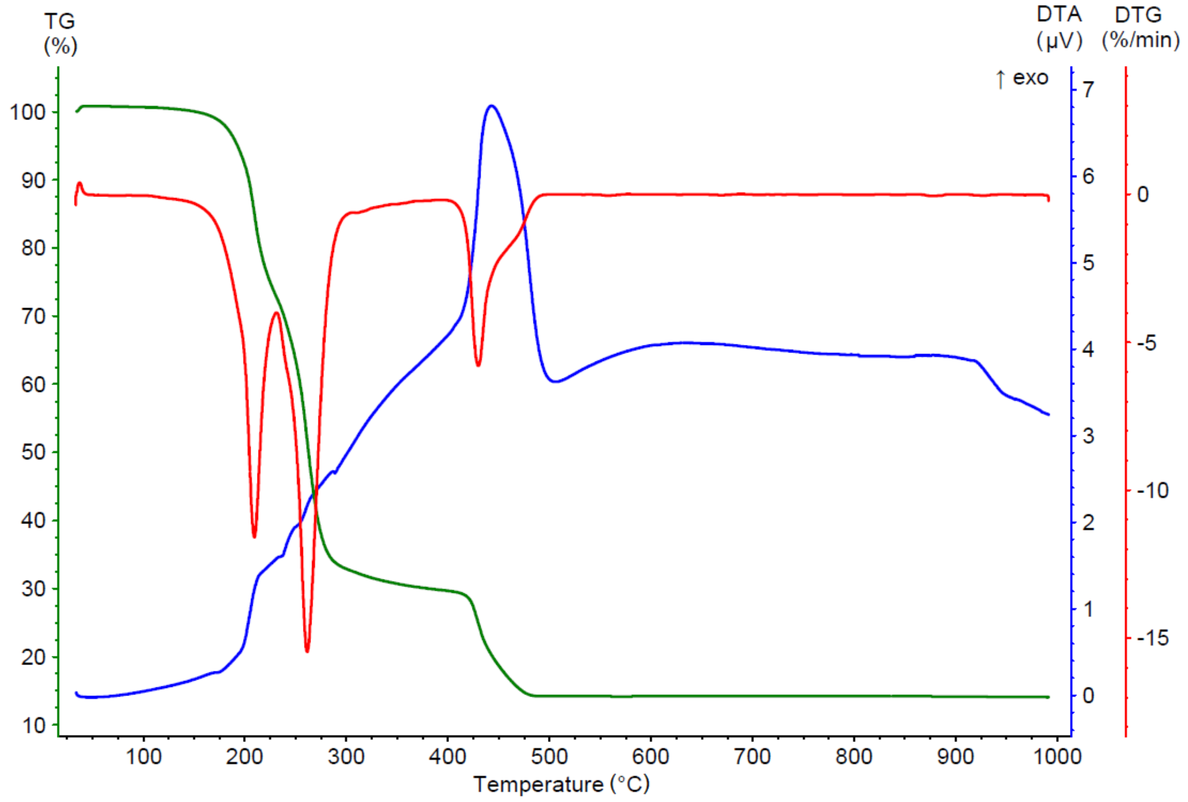

Figure S9. TG, DTA, and DTG curves for **2**.

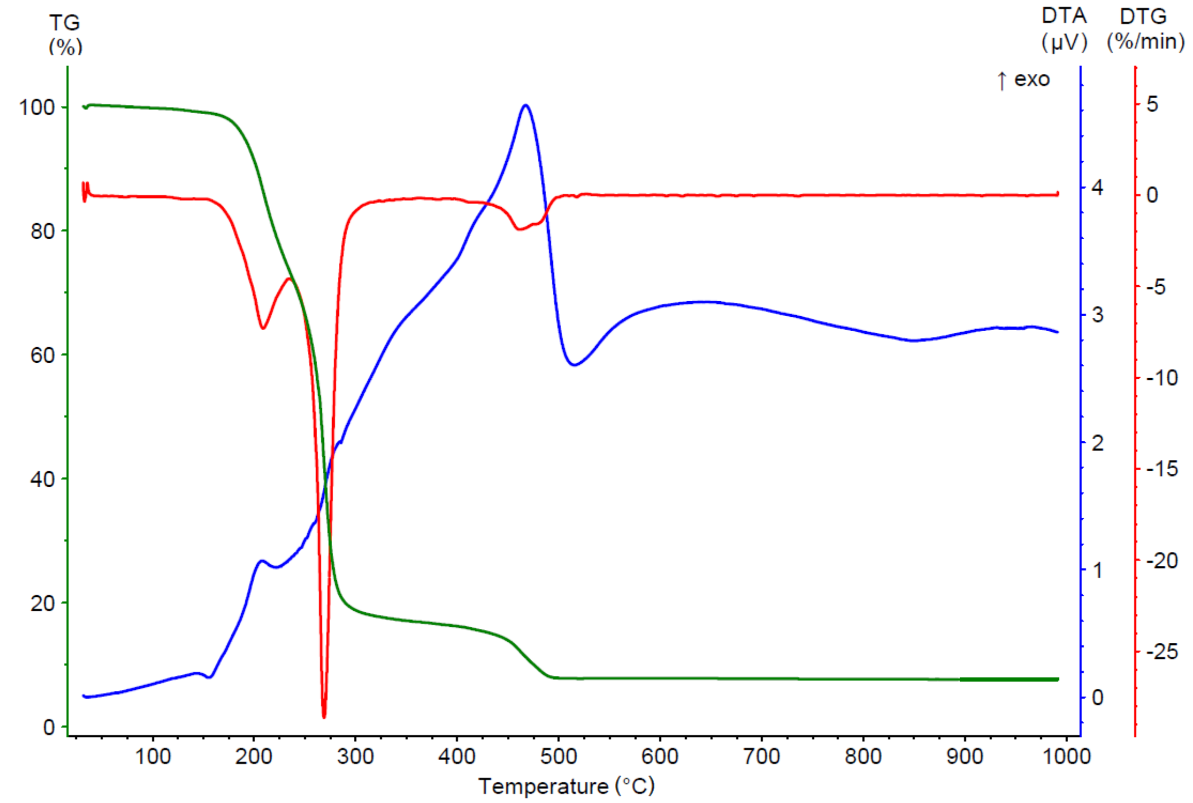

Figure S10. TG, DTA, and DTG curves for **3**.

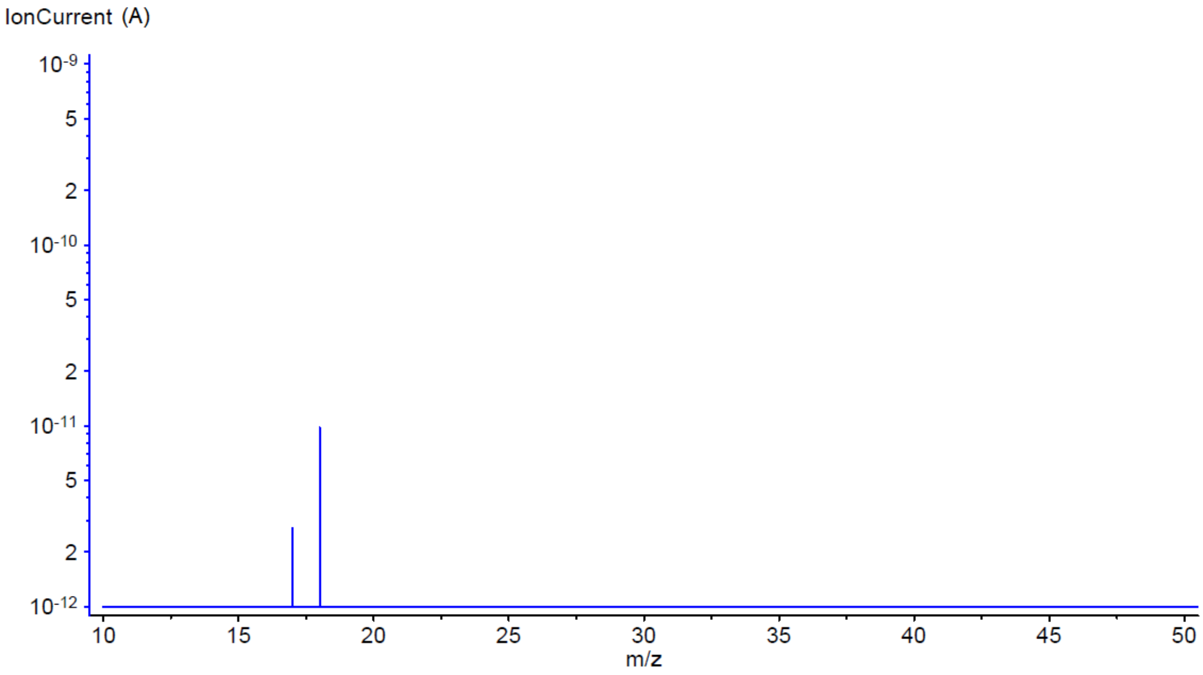

Figure S11. Mass spectrum of volatile products registered at 135 °C during thermal decomposition of **1**. The spectrum processing included background subtraction and the application of an automatic software correction for the carrier gas.

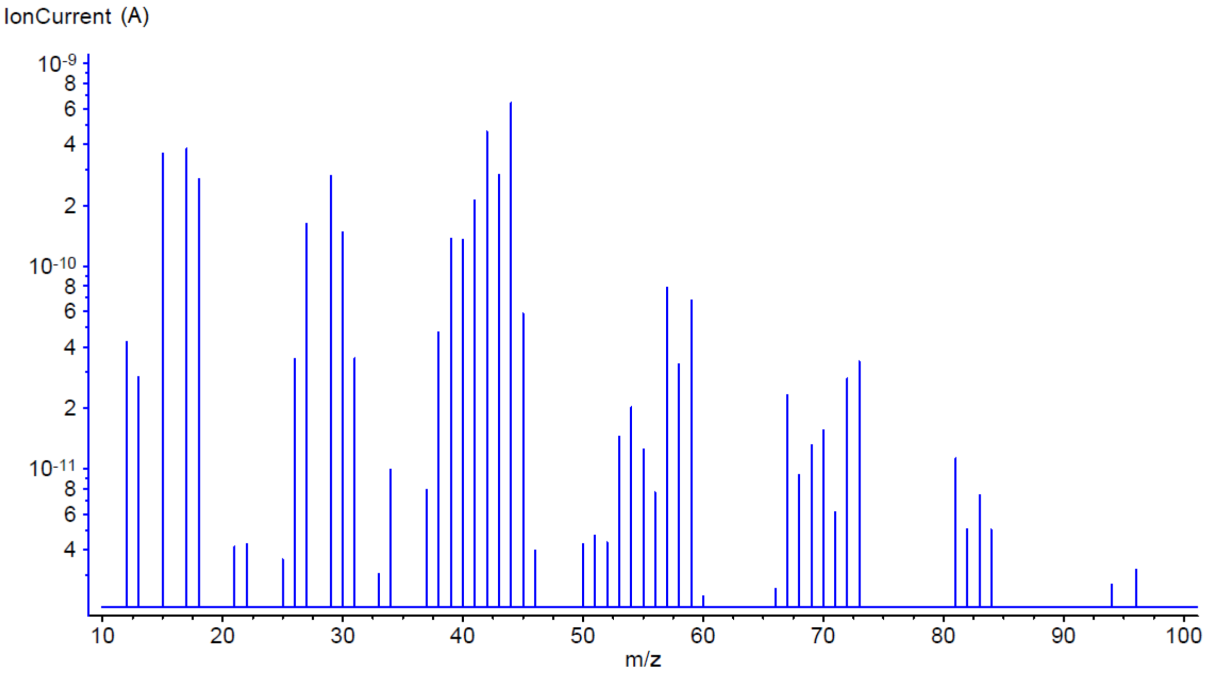

Figure S12. Mass spectrum of volatile products registered at 250 °C during thermal decomposition of **1**. The spectrum processing included background subtraction and the application of an automatic software correction for the carrier gas. The mass spectra of **2** and **3** registered at this temperature contain the same m/z signals.

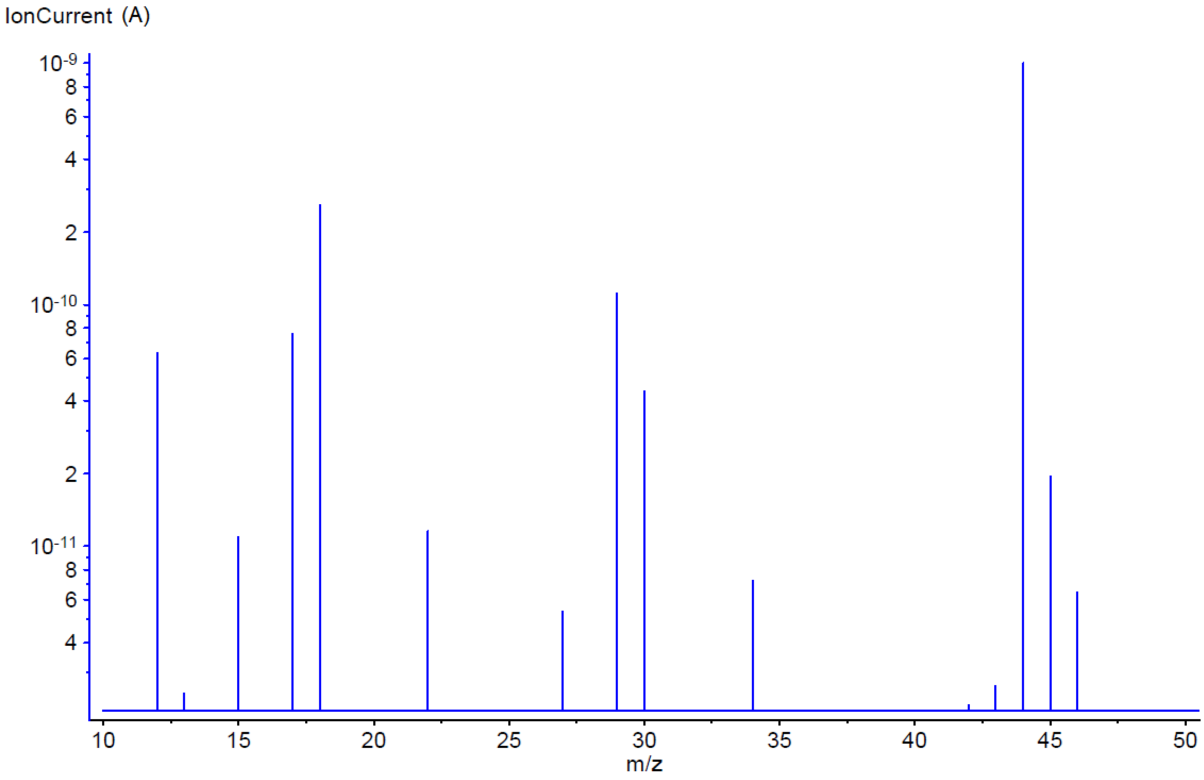

Figure S13. Mass spectrum of volatile products registered at 470 °C during thermal decomposition of **1**. The spectrum processing included background subtraction and the application of an automatic software correction for the carrier gas. The mass spectra of **2** and **3** registered at this temperature contain the same m/z signals.

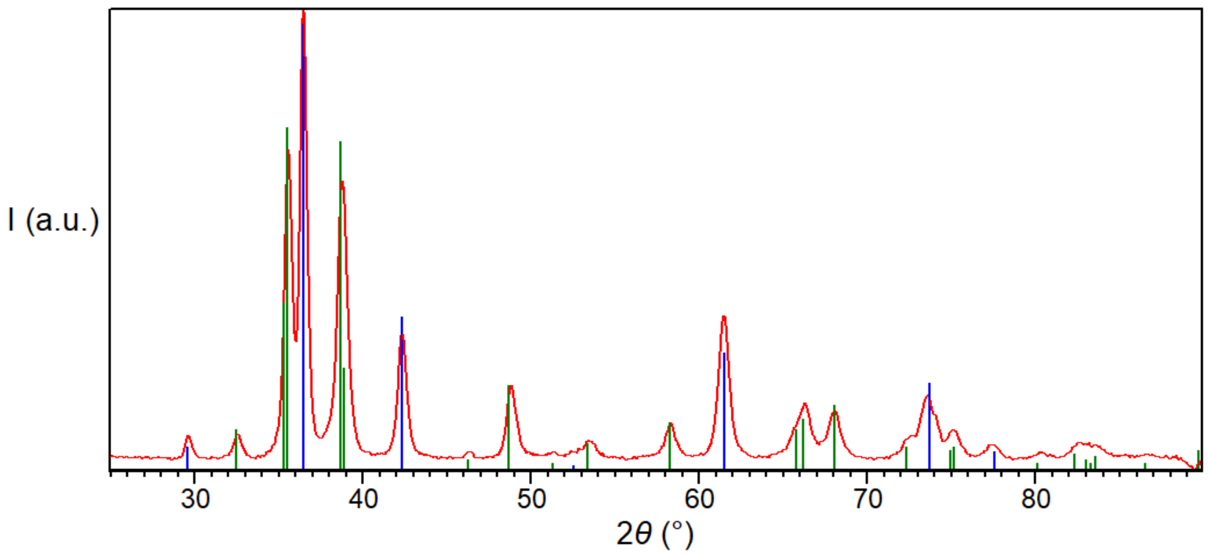

Figure S14. The XRPD pattern of the final product obtained after thermal decomposition of **1** (red) and reference patterns of tenorite PDF-2 code 00-005-0661 (green) and cuprite PDF-2 code 01-077-0199 (blue). The XRPD patterns of final products from thermal decompositions of **2** and **3** contain reflexes at the same 2θ angles.

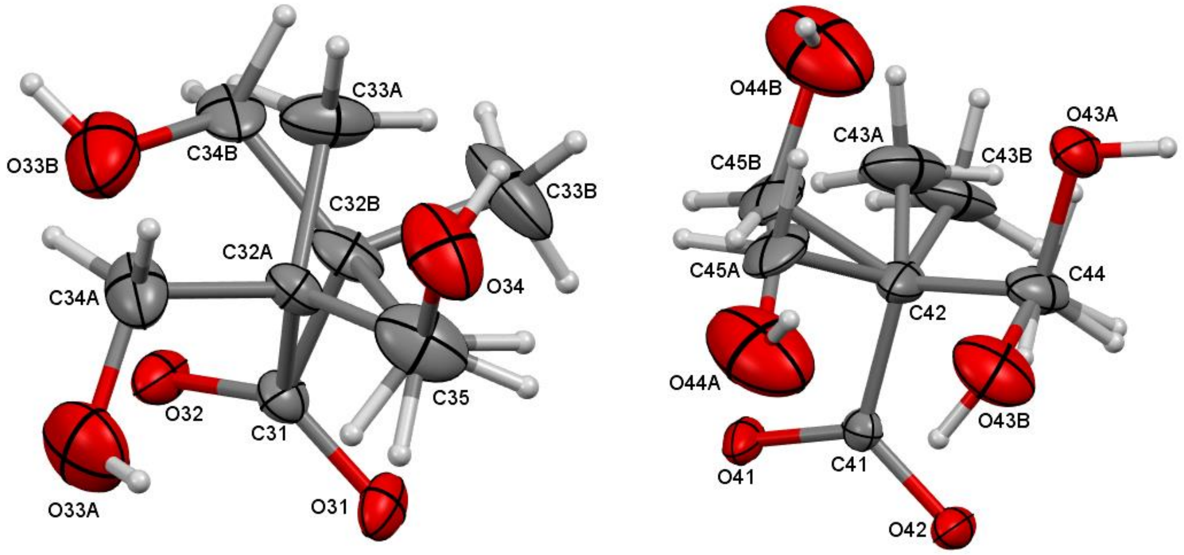

Figure S15. Disorder models of two dmp anions of **3**.

Table S1. Crystal data and structure refinement details for the studied compounds.

Compound	1	2	3
Empirical formula	$C_{22}H_{44}CuN_8O_9$	$C_{32}H_{60}Cu_2N_8O_{16}$	$C_{26}H_{48}Cu_2N_4O_{16}$
Formula weight	628.19	939.96	799.76
Crystal system	Triclinic	Triclinic	Triclinic
Space group	*P*-1	*P*-1	*P*-1
Unit cell dimensions			
a (Å)	6.36710(17)	10.1317(3)	10.7273(2)
b (Å)	10.8413(2)	10.4984(3)	11.6099(2)
c (Å)	21.2275(6)	11.4422(3)	16.2812(2)
α (°)	75.729(2)	65.146(2)	93.8260(10)
β (°)	85.889(2)	68.518(2)	105.8930(10)
γ (°)	75.047(2)	68.281(2)	115.0580(10)
Volume ($Å^3$)	1371.93(7)	993.39(5)	1727.36(5)
Z	2	1	2
Calculated density (Mg/m^3)	1.521	1.571	1.538
Absorption coefficient (mm^{-1})	1.691	2.023	2.184
F(000)	666	494	836
Crystal size (mm)	0.013 x 0.022 x 0.088	0.052 x 0.077 x 0.348	0.044 x 0.074 x 0.121
θ Range for data collection (°)	4.282 to 78.777	4.398 to 78.690	4.278 to 78.706
Index ranges	$-7 \leq h \leq 8$	$-10 \leq h \leq 12$	$-10 \leq h \leq 12$
	$-13 \leq k \leq 13$	$-12 \leq k \leq 13$	$-12 \leq k \leq 13$
	$-25 \leq l \leq 26$	$-13 \leq l \leq 14$	$-13 \leq l \leq 14$
Reflections collected / unique	36740/ 5610	20483/ 4054	34965/ 7073
R_{int}	0.0856	0.0338	0.0438
Completeness (%)	99.9	99.7	99.9
Min. and max. transmission	0.907 and 1.000	0.508 and 1.000	0.772 and 1.000
Data / restraints / parameters	5610 / 0 / 367	4054 / 0 / 268	7073 / 0 / 512
Goodness-of-fit on F^2	1.078	1.084	1.067
Final *R* indices [I>2σ(I)]	$R1$ = 0.0341	$R1$ = 0.0262	R1= 0.0399
	$wR2$ = 0.0951	$wR2$ = 0.0647	$wR2$ = 0.1126
R indices (all data)	$R1$ = 0.0406,	$R1$ = 0.0427,	R1= 0.0426
	$wR2$ = 0.0978	$wR2$ = 0.0746	$wR2$ = 0.1151
Largest diff. peak and hole (e•$Å^{-3}$)	0.400 and -0.353	0.629 and -1.085	1.251 and -0.754
